# Awareness of non-communicable diseases in women: a cross-sectional study

**DOI:** 10.1007/s00404-022-06546-9

**Published:** 2022-04-14

**Authors:** Fiona Irani, Eloïse Coquoz, Michael von Wolff, Norman Bitterlich, Petra Stute

**Affiliations:** 1 Department of Obstetrics and Gynecology, University of Bern, Bern, Switzerland; 2 Medizin and Service GmbH, Chemnitz, Germany; 3 Gynecological Endocrinology and Reproductive Medicine, University Women’s Hospital Inselspital, Friedbuehlstrasse 19, 3010 Bern, Switzerland

**Keywords:** Chronic non-communicable disease, Awareness, Lifestyle modification, Prevention, Public health

## Abstract

Chronic non-communicable diseases (NCD) are the major reason for death, morbidity, loss of independency and public health cost. NCD prevalence could be significantly reduced by adopting a healthy lifestyle. This cross-sectional cohort study (online survey) in 221 women aimed to assess NCD awareness, knowledge about NCD prevention and willingness to adopt a healthier lifestyle in women. Overall, NCD awareness level was quite high with, however, information mainly originating from lay media, probably being one reason for false estimations of age groups mainly affected by NCD, impact of NCD on quality of life, NCD mortalities, and the extent of NCD prevention by lifestyle interventions, respectively. Furthermore, also due to mainly lay media, half of women knew online NCD risk calculators, most of them would like to know their NCD risk, but only few had been offered NCD risk calculation by their physician. The mean threshold for willing to adopt a healthier lifestyle was a roughly calculated 37% 5–10 years risk to develop a certain NCD. Acceptance of non-pharmacological interventions for NCD prevention was high, however, major barriers for not implementing a healthier lifestyle were lack of expert information and lack of time. In conclusion, future public health strategies should focus on distributing better understandable and correct information about NCD as well as meeting the individuals’ request for personalized NCD risk calculation. Furthermore, physicians should be better trained for personalized NCD prevention counseling.

**Supplementary Information:**

The online version contains supplementary material available at 10.1007/s00404-022-06546-9.

## Introduction

In Western countries, life expectancy and thus the significance of non-communicable diseases (NCD) have increased [1, 2]. Nowadays, NCD are the major reason for death, morbidity, loss of independency and public health cost [3]. However, according to the WHO 30–50% of cancers and 80% of heart diseases, strokes and T2DM could be prevented or delayed by lifestyle changes [4, 5]. Lifestyle changes comprise physical activity, healthy diet, avoidance of tobacco and harmful amounts of alcohol. In 2013, the Swiss Department of Health initiated a nationwide strategy to reduce the individual and public health burden caused by NCD [4, 6]. Strategic approaches range from individual counseling [7, 8] to more generic ones like education about NCD (prevention). The aim of this cross-sectional cohort study was to assess NCD awareness, knowledge about NCD prevention and willingness to adopt a healthier lifestyle in women.

## Material and methods

### Study design

This was a cross-sectional cohort study. The study protocol was approved by the cantonal ethics committee (No Req-2017-00365).

### Study population

Women in Switzerland aged at least 18 years and speaking either German or French were included. Exclusion criteria were illiteracy and lack of internet access. Study participation was voluntary and anonymous. The questionnaire was sent to all female employees at the Department of Obstetrics and Gynecology Inselspital, members of two Swiss scientific societies, and, randomly, to all women of the University of Bern’s directory whose last name started with A, B or W, respectively. In addition, the online link was distributed via social media. Data collection was performed between July and August 2017.

### Questionnaire

The non-validated questionnaire comprised 30 questions (Q): four dichotomous questions, nine multiple choice questions with single (*n* = 2) and multiple (*n* = 7) answer options, one rating scale question, one constant sum question, five matrix table questions and two open-ended question (supplementary file 1). Questions addressed awareness level of NCD prevalence, NCD risk calculators and NCD prevention. Furthermore, questions assessed the subjects’ interest in NCD risk calculators and information level about NCD prevention, and the impact of both on individual lifestyle choices. Retrospective changes of the answers chosen were not possible. Furthermore, demographic characteristics were assessed to allow for subgroup analysis. The questionnaire was programmed on ‘my.unipark.com’.

### Classification of prevention category

There are three prevention levels [primary (I°), secondary (II°), tertiary (III°)] [4, 4, 6]. According to that a person can be either healthy (I° prevention), at risk for a certain disease (II° prevention), or affected by a certain disease (III° prevention). Each participant was assigned to a certain prevention group based on Q2 and Q28-30. Nine behavioral risk factors were assessed and weighed depending on their impact on the risk for developing a certain disease in a 60 years old 60 kg woman [9–12]. Obesity or dyslipidemia/statin therapy were scored with five points each. Overweight was scored with two points. All other risk factors were scored with one point [13–15]. Age was not taken into consideration. I° prevention was defined as having one point at maximum and not being affected by NCD. II° prevention was defined as having at least two points due to behavioral risk factors. If in Q2 the participant agreed to have had a certain disease she was assigned to category III° prevention.

### Classification of NCD awareness level

Q1, Q2 and Q4–Q7 were used to differentiate between moderate and high NCD awareness level. Questions’ importance for NCD awareness level classification was weighed so that the highest reachable score differed between questions. The relevance of Q5, Q6 and Q7 was rated to be highest (six points at maximum each). Q1 and Q4 were considered to be moderately relevant; maximal scores were two points and four points, respectively. Q2 was rated to have the least impact (three points at maximum). In detail, the point distribution for each question was as follows: Q5 lists six NCD. Their estimated impact on QoL was assessed on a 7-point scale (0 = don’t know, 1 = very little, 2 = little, 3 = some, 4 = moderately, 5 = a lot, 6 = severely; answer 0–3 = 0 points, answer 4–6 = 1 point; maximal score Q5 = 6 [16]). Q6 lists six NCD as major reason for death in Swiss women. The participant was asked to rank disease mortalities and to estimate absolute numbers of death per 100 women. 0.5 points were given if the individual NCD was ranked correctly or the attributable number of deaths estimated within a range of ± 25%, respectively (maximal score Q6 = 6) [1]. Q7 addresses the suspected preventability of NCD (not preventable = 0 points, preventable = 6 points). Q4 lists four age categories (1: < 45 years, 2: 45–64 years, 3: 65–84 years, 4: > 84 years) and the participant was asked to rate in which age category women are predominantly affected by NCD (age categories 1 = 0 points, 2 = 0 points, 3 or 4 = 2 points, 3 and 4 = 4 points, maximal score Q4 = 4). Q1 enquires about the term NCD (0 versus 2 points). Q2 lists nine NCD which were to be rated on a 4-point scale (1 = I don’t know, 2 = I have heard about it, 3 = I have been confronted with it by friends or family, 4 = I am/have been affected myself; maximal score Q2 = 3). As the maximum level was 27 points, the cut-off value between moderate (< 20) and high NCD awareness level (≥ 20) was set to 20 points corresponding to the third quartile (20.25 points). The point distribution was considered to best reflect the motivation for lifestyle changes (the higher the NCD awareness level the higher the motivation for lifestyle changes).

### Statistical analysis

Statistical analysis was performed using SPSS software (version 22.0). Frequencies and percentages were calculated for ordinal and nominal data. Chi-Square and exact Fisher’s test were used to determine the significance of any differences between rates. Descriptive means and standard deviations were calculated for ordinal data, differences in these were shown by Mann–Whitney-*U* test by two independent groups and by Kruskal–Wallis test by more than two independent groups. *p* values ≤ 0.05 were considered statistically significant (two-side). The aim was to recruit at least 100 participants to be able to calculate confidence intervals (CI) of ± 10% range.

## Results

### Cohort’s characteristics

221 in 250 participants fulfilled the inclusion criteria. Table 1 presents the cohort’s characteristics. Most subjects were below age 45, one fifth was within the age range 45–64 years. Based on personal history and NCD risk factors, two thirds belonged to category I° prevention (64.7%). BMI was 22.2 ± 3.1. 67.9% were childless. To most participants health was of moderate (35.7%) or high (62.9%) importance. Worries about personal health were assessed on a 11-point Likert scale. The median 3.0 was set as threshold for having had few (≤ 3 points) or frequent health worries (≥ 4 points) during the previous month. 47.1% of participants frequently worried about their health status. Intergroup comparisons revealed a significant impact of age and NCD prevention level on the frequency of health worries (age ≤ 44 years 48.8% vs. age 45–64 years 35.4% vs. age ≥ 65 years 77.8%, *p* < 0.048; NCD prevention I° 39.9% vs. II° 59.9% vs. III° 61.0%, *p* = 0.015). The percentage of women speaking German (52%) or French (48%) was equally distributed. 57% of participants had the highest educational degree (university). Job occupation was mostly part-time (38.9%), full-time (28.5%) or being a trainee (36.7%).

**Table 1:** Characteristics of the cohort (*n* = 221)

Characteristic	Number (%)
Age	
18–39 years	164 (74.2)
40–59 years	48 (21.7)
60–74 years	7 (3.2)
≥ 75 years	2 (0.9)
Language	
German	115 (52.0)
French	106 (48.0)
Highest level of education	
Primary school	13 (5.9)
High school	76 (34.4)
University	126 (57.0)
Others	6 (2.7)
Occupation/job	
Trainee	81 (36.7)
Unemployed	7 (3.2)
Employed, full-time	63 (28.5)
Employed, part-time	86 (38.9)
Housewife	33 (14.9)
Retired	13 (5.9)
Having children	
Yes	71 (32.1)
No	150 (67.9)
Risk factors for chronic non-communicable diseases	
Overweight/obesity	35 (15.8)
Tobacco (active smoker)	18 (8.1)
Tobacco (passive smoker)	12 (5.4)
Alcohol (daily)	5 (2.3)
Alcohol (occasionally)	75 (33.9)
Fast food	19 (8.6)
Low physical activity	74 (33.5)
Dyslipidemia / hypercholesterinemia	16 (7.2)
Arterial hypertension	6 (2.7)
Hyperglycemia	3 (1.4)
Other	79 (35.7)
Prevention level regarding chronic non-communicable diseases	
Primary prevention level	143 (4.7)
Secondary prevention level	37 (16.7)
Tertiary prevention level	41 (18.6)

### Awareness level of chronic non-communicable diseases

61.1% of participants had not heard of the term “non-communicable disease” before. However, all diseases defined as NCD were well known but COPD. When differentiating between moderate (< 20 points) and high (≥ 20 points) NCD awareness level, 42.1% fell into the first category. Language origin (*p* = 0.029), education (*p* = 0.006), significance of health status (*p* = 0.022) and having children (*p* = 0.042) had a significant impact on NCD awareness level. Accordingly, NCD awareness level was significantly more likely to be high in French speaking, highly-educated mothers to whom health was of great importance (data not shown). Sources of information were mainly environment (85.5%), media (newspaper 63.8%, TV 54.8%) but rarely physicians (21.3%). Newspapers were significantly more often reported as source by older women (*p* = 0.016), women with high NCD awareness level (*p* < 0.001) and women to whom their status was very important (*p* = 0.028) (data not shown).

38.5% thought that women aged 45–64 years were predominantly affected by NCD while only 4.5% thought that this was true for women at age 65 + . Most participants estimated the impact of cancer (90.9%), dementia (90.5%), CVD (85.1%), musculoskeletal (87.8%) and pulmonary diseases (79.2%) on QoL was at least moderate. The strongest impact on QoL was assigned to dementia with 53.4% rating its impact as maximal. In contrast, T2DM was estimated to have a lower impact on QoL as 83.2% rated its impact to range between “some” and “a lot”. Disease burden was rated significantly higher by younger and less educated women (supplementary table 1). Disease mortalities were asked to be ranked with also estimating the mean absolute numbers of death per 100 Swiss women (Table 2). CVD were ranked as top killer accounting for 28.2 ± 12.9% of deaths, followed by cancer (25.8 ± 10.5%). Less than 10% of deaths were attributed to pulmonary diseases, T2DM, dementia and musculoskeletal diseases. When differentiating between participants who had only heard of the disease and those who were affected by it (e.g., personal or family history), the estimated mortality rate significantly differed for CVD and musculoskeletal diseases (Table 2).

**Table 2:** Estimated mortality rate (number of deaths per 100 women in Switzerland) for major non-communicable diseases

Non-communicable disease	(a) Estimated mortality rate (number of deaths per 100 women)	(b) Estimated mortality rate (number of deaths per 100 women) if only heard of the disease	(c) Estimated mortality rate (number of deaths per 100 women) if affected by the disease	*p* value for comparison of (b) and (c)
CVD (e.g. myocardial infarction)	28.2 ± 12.9	25.0 ± 13.5	30.3 ± 12.2	< 0.001
Cancer	25.8 ± 10.5	Comment: no statistical differentiation was made as Q2 and Q6 addressed either all cancer types together (Q6) or separately (Q2)		
Pulmonary diseases	9.2 ± 6.1	9.6 ± 6.8	9.8 ± 5.3	0.307
Diabetes mellitus	8.9 ± 6.6	8.9 ± 7.7	9.0 ± 6.2	0.376
Dementia	6.4 ± 5.4	6.4 ± 5.5	6.4 ± 5.4	0.861
Musculoskeletal diseases	5.6 ± 5.7	6.8 ± 5.8	5.2 ± 5.6	0.015
Other	15.5 ± 12.6	Not applicable		

*CVD* cardiovascular disease

### Knowledge about chronic non-communicable disease prevention

93.2% were convinced they could prevent/delay NCD development by lifestyle interventions. However, the realistic extent of NCD prevention by lifestyle interventions (40–60%) was correctly estimated by only 46.2%. Healthy (I° prevention group 20.3%), childless (19.3%) and full-time working (24.2%) women were significantly more optimistic (estimated extent of NCD prevention by lifestyle interventions > 60%). In contrast, women affected by NCD (III° prevention group 53.7%), women with children (47.9%) and housewives/retired women (47.4%) were more pessimistic (estimated extent of NCD prevention by lifestyle interventions < 40%). Interestingly, age, education, and frequency of worries about health did not have a significant impact on the estimated extent of NCD prevention by lifestyle interventions (< 40%, 40–60%,  > 60%) (data not shown).  > 90% perceived the following measures to effectively reduce NCD development: physical activity, healthy nutrition, tobacco avoidance, stress reduction and spending time in nature. Two thirds of participants believed that some alcohol, living in the countryside and chemoprevention (prescription medication) would prevent NCD. Sugar, salt and meat consumption were negatively associated with NCD prevention.

To personalize NCD prevention recommendations (online) risk calculators can be applied. Based on lifestyle, personal and family history, clinical examination and blood tests, risk calculators may estimate the individual chance to develop a certain disease within a defined time period. This information allows physician and patient to develop an individual health prevention and promotion strategy focusing on self-empowerment and lifestyle. In our cohort, 43% had heard about risk calculators before. Knowledge about risk calculators was not significantly affected by age, language, having children, NCD prevention and awareness level, education, significance of health status and frequency of being worried about health. Media (newspapers/magazines (54.7%), TV (35.8%), internet (32.6%)) were the main sources of information about risk calculators. School (35.6%) and relatives/friends (30.5%) were also important. In contrast, physicians played a minor role (14.7%). Accordingly, only 5.3% reported their individual NCD risk had been calculated by their physician (CVD, cancer). When being asked which NCD risk would be of personal interest the following NCD categories were reported: cancer (68.8%), CVD (62.9%), musculoskeletal diseases, dementia (51.6%), T2DM (40.3%), and pulmonary diseases (29.9%). Frequencies were not significantly affected by age, having children, NCD prevention level, education, significance of health status, frequency of being worried about health, or the subjectively estimated impact of a specific NCD on QoL. However, German speaking women were significantly more often interested in knowing their personal NCD risk than French speaking women (*p* = 0.010). Similarly, women with moderate NCD awareness level significantly more often wanted to know their personal NCD risk than women with good NCD awareness level (*p* = 0.025). Only 15.8% had no interest in personal NCD risk.

### Willingness to adopt a healthier lifestyle

Knowing anything (mental level) does not necessarily transform into changing habits (action level). Thus, one of the biggest challenges is to find out when someone’s personal threshold is reached to put knowledge into action. Therefore, women were asked which 5–10 years NCD risk (in %) would be high enough to significantly change lifestyle habits. Mean threshold was 36.8 ± 20.8%. While age, having children, NCD prevention and awareness level, education, and frequency of being worried about health did not have a significant impact on this number, language and significance of personal health status did. French-speaking women and those to whom health was highly important chose a significantly lower threshold for becoming active (German-speaking 40.1 ± 21.2% vs. French-speaking 33.2 ± 19.7%, *p* = 0.008; moderate 42.4 ± 20.4% vs. high importance of health 33.0 ± 19.7%, *p* = 0.001). To estimate the willingness to adopt a healthier lifestyle, participants were confronted with three fictitious medical scenarios representing I°, II° and III° NCD prevention (Table 3). Various lifestyle interventions were offered to choose from and the degree of willingness was assessed. Overall, the willingness to adopt a healthier lifestyle to prevent/delay NCD development was high. Some of the recommended actions were already implemented in daily life by at least one third of participants (not smoking, reduced intake of sweetened beverages, daily physical activity). NCD chemoprevention was the least accepted suggested action. Overall, the willingness to change lifestyle was merely affected by NCD prevention level. However, the willingness for physical activity was lower if the subject was sicker (NCD prevention III°) (data not shown).

**Table 3:** Willingness to change lifestyle habits depending on the prevention level of a fictitious medical scenario (*n* = 221)

Willingness to change lifestyle habits depending on prevention level of fictitious medical scenario [*n* (%)]			
Lifestyle intervention	I° (%)	II° (%)	III° (%)
Smoking cessation			
Immediately	59 (26.7)	74 (33.5%)	81 (36.7%)
Maybe	20 (9)	11 (0.5%)	5 (2.3%)
Not willing	1 (0.5)	1 (0.5%)	0
Not applicable	140 (63.3)	131 (59.3%)	134 (60.6%)
Alcohol avoidance			
Immediately	96 (43.4)	117 (52.9%)	152 (68.8%)
Maybe	70 (31.7)	60 (27.1%)	28 (12.7%)
Not willing	16 (7.2)	7 (3.2%)	0
Not applicable	36 (16.3)	33 (14.9%)	38 (17.2%)
Physical activity at least 30 min per day			
Immediately	93 (42.1)	111 (50.2%)	120 (54.3%)
Maybe	29 (13.1)	14 (6.3%)	6 (2.7%)
Not willing	1 (0.5)	0	1 (0.5%)
Not applicable	98 (44.3)	96 (43.4%)	94 (42.5%)
Chemoprevention (prescription medication)			
Immediately	25 (11.3)	61 (27.6%)	119 (53.8%)
Maybe	107 (48.4)	98 (44.3%)	73 (33.0%)
Not willing	62 (28.1)	41 (18.6%)	8 (3.6%)
Not applicable	10 (4.5)	10 (4.5%)	15 (6.8%)
Healthy diet/nutritionist counseling			
Immediately	126 (57)	151 (68.3%)	155 (70.1%)
Maybe	38 (17.2)	17 (7.7%)	7 (3.2%)
Not willing	1 (0.5)	0	0
Not applicable	56 (25.3)	52 (23.5%)	59 (26.7%)
Reduced intake of sweetened beverages			
Immediately	96 (43.4)	102 (46.2%)	106 (48.0%)
Maybe	8 (3.6)	11 (5.0%)	7 (3.2%)
Not willing	2 (0.9)	0	0
Not applicable	115 (52.0)	108 (48.9%)	108 (48.9%)
Stress management/counseling			
Immediately	91 (41.2)	136 (61.5%)	168 (76.0%)
Maybe	90 (40.7)	53 (4.0%)	26 (11.8%)
Not willing	7 (3.2)	5 (2.3%)	2 (0.9%)
Not applicable	21 (9.5)	22 (10.0%)	23 (10.4%)
Participation in breast cancer screening program			
Immediately	103 (46.6)	132 (59.7%)	158 (71.5%)
Maybe	53 (24.0)	42 (19%)	21 (9.5%)
Not willing	16 (7.2)	9 (4.1%)	5 (2.3%)
Not applicable	37 (16.7)	32 (14.5%)	35 (15.8%)
Participating in colorectal cancer screening program			
Immediately	75 (33.9)	119 (53.8%)	165 (74.7%)
Maybe	101 (45.7)	76 (34.4%)	37 (16.7%)
Not willing	21 (9.5)	7 (3.2%)	2 (0.9%)
Not applicable	13 (5.9)	12 (5.4%)	13 (5.9%)

Comment: not applicable = already following advice

In contrast to our study offering several lifestyle interventions this might not be true in reality. Therefore, subjects were asked if information on NCD prevention measures was sufficiently available and who should be the main information source. For 59.3%, information availability on NCD prevention measures was insufficient. In particular, women below age 45 were dissatisfied (65.9%). Main information sources should be school (61.5%), physicians (57.0%), media (newspaper 55.2%, TV 41.6%), radio (27.6%), public authorities (33.9%), and health insurances (32.1%). Besides lack of information, other barriers might also stop the individual from adopting a healthier lifestyle. Lack of time was the biggest barrier (lack of time for physical activity 35.7%, and for healthy diet 23.5%). Also, 29.0% did not know what to do. In contrast, 24.9% were not convinced that lifestyle changes were necessary. Interestingly, cost for healthy diet and sports did not seem to be a major issue. Still, 71.5% were convinced the public health system should spend more money for prevention.

## Discussion

In this cross-sectional cohort study in mostly healthy, well-educated, health-sensitive, midlife women, we found that (1) overall NCD awareness level was quite high with (2) information mainly originating from lay media, probably being one reason for (3) false estimations of age groups mainly affected by NCD, impact of NCD on QoL, NCD mortalities, and the extent of NCD prevention by lifestyle interventions, respectively. Furthermore, (4) also due to mainly lay media, half of women knew online NCD risk calculators, (5) most of them would like to know their NCD risk, but (6) only few had been offered NCD risk calculation by their physician. Changing lifestyle habits is a challenge. Therefore, one important prerequisite is to be convinced that this (at least in the beginning) discomfort indeed may have a positive impact on oneself health. (7) The mean threshold for willing to adopt a healthier lifestyle was a roughly calculated 37% 5–10 years risk to develop a certain NCD. (8) Overall, acceptance of non-pharmacological interventions for NCD prevention was high, however, (9) major barriers for not implementing a healthier lifestyle were lack of expert information and time.

NCD account for 86.3% of disease burden [17]. In our study, the majority was convinced that mainly subjects aged between 45 and 64 years were affected by NCD. However, this was a misjudgment as disease burden from NCD measured in DALYs and classified by age group was reported to be 38.5% (≥ 70 years), 31.9% (50–69 years), 26.8% (15–49 years), 1.7% (5–14 years), and 1.1% (< 5 years), respectively [18]. Similarly, while our cohort thought dementia had the strongest negative impact on QoL, NCD disease burden was found to be highest for CVD, followed by cancer, musculoskeletal disorders, diabetes and endocrine diseases, chronic respiratory disease, and neurological disorders [19]. Approximately 42% of all NCD deaths globally occur before the age of 70 years [20]. Most participants in our study correctly assigned CVD and cancer to the top killers corresponding to WHO data (deaths from NCD: CVD 27.3%, cancer 10.2%, dementia 4.7%, chronic respiratory disease 3.2%) [21]. While multiple studies on NCD risk factors have been published, only few studies have investigated the awareness of and knowledge about NCD risk factors and NCD prevention in (healthy and diseased) populations. In our study, most participants were convinced they could prevent/delay NCD development by lifestyle interventions. Similarly, in a Scandinavian survey, smoking was a well-known risk factor for cancer. However, awareness was quite low (< 50%) for low fruit and vegetable intake and alcohol consumption as risk factors [22]. NCD awareness level was found to be even lower in less developed countries. In Uganda less than 50% of community health workers thought that T2DM was preventable [23]. In Rwanda, a survey in HIV infected people revealed that 65% had never heard the term “stroke”. Only 22% had information on NCD prevention [24]. Women from rural regions in India reported a high awareness level of smoking having a negative impact on health. Still, almost one half did not know the diseases that could be caused by smoking. Nearly 60% of women had never heard of heart attack or stroke. Knowledge was even worse about breast (27.3%) and cervical cancer (11.5%), respectively, corresponding to very low prevalence of preventive physical examinations by health care providers (0.9% with ever breast examination, 1.3% with gynecologic checkup within the past 5 years) [25].

NCD risk management can be individualized by, e.g. using NCD risk calculators [7, 8]. Although most women in our study were interested in knowing their NCD risk, only few had been offered an NCD risk calculation by their physician. Thus, our survey supports previous studies showing people find NCD risk calculators helpful [26]. Yet, using NCD risk calculators without professional guidance may lead to false conclusions [26–28] strengthening the physician’s role. In our previous cross-sectional study in primary care providers most physicians were aware of NCD risk calculators [29]. However, still several barriers emerged, e.g. lack of time during counselling, lack of risk calculators that cover more than just one NCD, and uncertainty about which NCD risk calculator to use as there are several available. The latter barrier has been addressed by providing an overview of validated risk calculators for each NCD [7, 8]. NCD risk calculators transform the calculated risk to develop a certain NCD within a defined time period into risk categories (e.g. low, medium, high). Still, the actual risk number in % that defines a risk category may differ between NCD and risk calculation model. For example, within the Framingham Heart Study individuals with a 10-year risk of a CVD event > 20% were considered to have a high global CVD risk [30]. In contrast, the GAIL model for calculating breast cancer risk defined women with a calculated 5 years risk > 1.7% to be at increased risk [31]. Defining risk categories overcomes this issue and may help physicians to provide the patient with adequate and standardized recommendations. However, persuading and motivating a patient to put the recommended lifestyle changes into sustainable actions remain one of the biggest challenges. This is why we asked about the subjective threshold for willing to adopt a healthier lifestyle. In our cohort this was a roughly calculated 37% 5–10 years risk to develop a certain NCD. This number was higher than the lower threshold of most “high risk” categories of NCD risk calculators. We can only speculate that such a discrepancy between objective and subjectively perceived NCD affects individual willingness for lifestyle modification [32].

Overall, in our study, the willingness to adopt a healthier lifestyle to prevent/delay NCD development was high. However, as the three medical scenarios on I°, II° and III° prevention level were hypothetical it remains questionable as to whether participants would actually engage into lifestyle changes. Previous studies in II° prevention have shown conflicting results. In a cross-sectional study, patients with CHD were not ready to change their health-related behaviors [33]. Similarly, a cross-sectional study in diabetics revealed that almost 60% did not follow a diet plan [34]. In contrast, a 3 months RCT in patients following TIA and stroke observed a clinically significant effect in reported exercise and diet behavior in the intervention group that received additional advice, motivational interviewing and telephone support to change health behavior [35]. Similarly, in another 2 years RCT women with CHD assigned to a comprehensive lifestyle self-management program showed significantly greater improvements compared to the usual care control group in respect to BMI, angina symptoms, and QoL, and a tendency for a greater reduction in blood pressure-lowering medications [36]. However, a second 5 years RCT in patients with CHD did not report significant differences between groups in respect to blood pressure, serum lipids, BMI, angina frequency, or activity restriction at 5 years when comparing a 2 years personal health promotion program to a usual care control group. Interestingly, significant group differences for exercise frequency and diet changes were only seen during the 2 years program but had worn off afterwards [37].

Several promoting factors have been identified increasing the likelihood to adhere to lifestyle changes, e.g. focusing on improved QoL (not life expectancy) [38], having a higher risk profile for a certain NCD [33], higher income [33], knowledge [39], education flexibility in terms of timing post-event and modes of delivery [34, 40], resistant attitudes without pessimism and helplessness [41, 42], self-efficacy [43] and family intervention [44], respectively. In contrast, the number of barriers to adopt a healthy lifestyle seems overwhelming. This was also found in our survey. Barriers can be differentiated into several categories, e.g., lack of professional support, temptations and treats, unhelpful social contacts, personal problems [45], social and economic factors, therapy-, patient-, health system-, and condition-related factors [46, 47]. Obviously, these barriers interact and influence each other [46]. Accordingly, a recent review on barriers for physical activity in older adults listed poor health, lack of time and motivation/interest, tiredness, environment (e.g. safe place, weather), self-consciousness about physical appearance, and fear of injury as the dominating barriers [48]. Another barrier may be the distrust in its efficacy. This opinion may be supported by a recent meta-analysis in patients with TIA or stroke revealing that lifestyle interventions significantly reduced systolic blood pressure but had no effect on CVD mortality, diastolic blood pressure, or total cholesterol [49]. In contrast, a 2 years RCT in women with CHD found that a comprehensive, intensive, multicomponent lifestyle self-management program (diet change, stress management, exercise, group support, smoking cessation) had a (significantly) beneficial effect on CVD risk factors, CVD symptoms and QoL [36]. Similarly, a 2 years RCT in men with CHD found that a multicomponent lifestyle intervention (diet change, exercise, smoking cessation, education, psychosocial support) resulted in a 5 years CHD calculated relative risk reduction by 22% [50]. The importance of a multicomponent lifestyle intervention approach was supported by a 3 months study in patients with CHD showing that improvements in dietary fat intake, exercise, and stress management were individually, additively and interactively related to coronary risk and psychosocial factors [51]. Several strategies have been developed to overcome these barriers, either targeting the patient [52, 53], his/her surroundings/family [44, 54], the physician [46, 55] and/or the public health system [54, 56–58] . Yet, the success rates still need to be evaluated [59]. So far, there are no studies comparing strategies and their success rates between different countries and health care systems [59, 60].

Clearly, our study has some limitations. Due to the study design we only collected anonymous data which prevented us from correlating the results to patient data. As we mostly included well educated women we cannot generalize them to men or less educated cohorts. Thus, future studies should implement additional strategies to reach people from low socioeconomic status, e.g. social media, eHealth (digital health applications), operational health management. We constructed a questionnaire which was not validated. However, in contrast to the Heart Disease Fact Questionnaire assessing CHD risk knowledge in people with diabetes by applying dichotomous questions only [61], we used multiple question types thereby reducing the error of measurement by guessing. We decided to construct our score based on medical knowledge also taking the three prevention levels into account. Thereby, we intentionally did not focus on, e.g. self-efficacy [62], but focused on uncovering misconcepts and information gaps which, in our opinion was especially helpful, as a certain level of disease threatening seems to be an important trigger for lifestyle changes [63]. However, the strength of this survey was the combined assessment of NCD awareness, knowledge about NCD, willingness and barriers to adopt a healthy lifestyle. Furthermore, by classifying participants by prevention level and language (cultural) background we were able to assess differences for those outcomes. For example, healthy women showed more readiness to preventive behaviors, and younger women had more interest in receiving further information about NCD (prevention).

## Conclusion

NCD prevalence could be significantly reduced by adopting a healthy lifestyle. Therefore, international public health care systems have developed several strategies to help people transforming their lifestyles. However, success rates are mostly low despite a quite high NCD awareness level (at least in Western countries). Yet, there is a lack of correct information about the prevalence, impact and prevention of specific NCD. Thus, future public health strategies should focus on distributing better understandable and correct information about NCD as well as meeting the individuals' request for personalized NCD risk calculation. Furthermore, physicians should be better trained for personalized NCD prevention counseling.

## Supplementary Information

Below is the link to the electronic supplementary material.

Supplementary file1 (DOCX 16 KB)

Supplementary file2 (DOCX 63 KB)

## Abbreviations

I°: Primary (prevention level)
II°: Secondary (prevention level)
III°: Tertiary (prevention level)
BMI: Body mass index [kg/m^2^]
CHD: Coronary heart disease
COPD: Chronic obstructive pulmonary disease
CVD: Cardiovascular disease(s)
DALYs: Disability-adjusted life years
HIV: Human immunodefficiency virus
NCD: Non-communicable disease
T2DM: Type 2 diabetes mellitus
TIA: Transient ischemic attack
QoL: Quality of life
Q: Question (of the survey)
RCT: Randomized-controlled trial
WHO: World Health Organization
