# Supplementary material for: Awareness of non-communicable diseases in women: a cross-sectional study
Source: Arch Gynecol Obstet. 2022 Apr 14;306(3):801–10. doi: 10.1007/s00404-022-06546-9 (PMC9411077; doi:10.1007/s00404-022-06546-9)
Supplement: Supplementary file 1 — Supplementary file1 (DOCX 16 KB) [file 404_2022_6546_MOESM1_ESM.docx]

| **Estimated burden of disease** | **Mean**  **(min = 6, max = 12)** | **p-value** |
| --- | --- | --- |
| Language  German  French | 9.52  9.53 | 0.902 |
| Prevention level  primary  secondary  tertiary | 9.66  9.59  8.98 | 0.051 |
| Age  ≤44y  45–64y  ≥65y | 9.75  9.12  7.56 | <0.001 |
| Education  e.g. university  e.g. high school  e.g. primary school | 8.29  9.34  9.77 | 0.004 |
| With children  Childless | 9.32  9.62 | 0.259 |
| Occupation  Trainee  Unemployed  Employed (full-time)  Employed (part-time)  Housewife or retired | 9.98  9.29  9.63  9.39  8.47 | 0.088 |
| Good knowledge  Average knowledge | 9.12  10.08 | <0.001 |
| Health status significance  high significance  other attitudes | 9.20  9.70 | 0.028 |
| Worries about health  worry often  worry seldom | 9.65  9.41 | 0.232 |
