# Supplementary material for: Awareness of non-communicable diseases in women: a cross-sectional study
Source: Arch Gynecol Obstet. 2022 Apr 14;306(3):801–10. doi: 10.1007/s00404-022-06546-9 (PMC9411077; doi:10.1007/s00404-022-06546-9)
Supplement: Supplementary file 2 — Supplementary file2 (DOCX 63 KB) [file 404_2022_6546_MOESM2_ESM.docx]

Supplementary file 1: Questionnaire

Inclusion criteria (all have to apply)

- Age ≥ 18 years
- Female
- Residence in Switzerland

**Part A**

During the last century, the prevalence of so-called chronic non-communicable diseases (NCD) such as cardiovascular diseases and cancer has increased. About one in four persons suffers from NCD. NCD generate 80% of the direct health cost. In 2013, the Federal Office of Public Health in Switzerland decided to develop strategies to improve this situation. One of these strategies aims to elevate the awareness and individual responsibility.

The Department of Obstetrics and Gynaecology aims to assess NCD awareness in women and also their willingness to take preventive measures against NCD. Thank you for answering the following questions as precisely as possible. Your answers will be analysed confidentially and anonymously.

Question 1: Have you ever heard the term «Non-Communicable Diseases» (NCD)?

- Yes
- No

*Comment: NCD is a generic term for diseases that cannot be transmitted from one person to another. The six most relevant NCD responsible for about two thirds of overall mortality are: 1) cardiovascular diseases (e.g., coronary heart disease, heart attack, stroke), 2) cancer (the most common in women: breast cancer, lung cancer, colon cancer), 3) type 2 diabetes mellitus (T2DM), 4) chronic lung diseases (e.g., chronic obstructive pulmonary disease, COPD), 5) musculoskeletal diseases (e.g., osteoporosis, osteoarthritis, back pain), and 6) dementia.*

Question 2: How familiar are you with the following diseases? (multiple answers possible)

|  | I don’t know it | I have heard about it. | I have been confronted with it through friends or family | I am / have been affected by it myself |
| --- | --- | --- | --- | --- |
| Heart attack / coronary heart disease |  |  |  |  |
| Stroke / cerebral vascular disease |  |  |  |  |
| Breast cancer |  |  |  |  |
| Lung cancer |  |  |  |  |
| Colon cancer |  |  |  |  |
| Diabetes mellitus |  |  |  |  |
| Chronic lung diseases (e.g., chronic obstructive pulmonary disease (COPD)) |  |  |  |  |
| Musculoskeletal disease (e.g., osteoporosis, osteoarthritis, back pain) |  |  |  |  |
| Dementia |  |  |  |  |
| Others: _____ |  |  |  |  |

Question 3: Where did you get the information about these diseases from? (multiple answers possible)

- My physician
- Family/friends
- Newspapers or (health-) magazines
- TV
- School / other educational activities
- Personal active search (sources _____)
- Other: _____

Question 4: In your opinion, which age classes in women are mostly affected by NCD? (multiple answers possible)

- < 45 years
- 45 - 64 years
- 65 – 84 years
- ≥ 85 years

Question 5: For each disease presented in the table, please rate how strong their impact on the quality of life in affected people is.

*Comment: Quality of life can be impaired by a disease in several ways, e.g., by the need to regularly measure body functions (e.g., blood pressure, sugar levels) or take medication, by less mobility, chronic pain.*

|  | No opinion | Very little | Little | Some | Moderately | A lot | Severely |
| --- | --- | --- | --- | --- | --- | --- | --- |
| Cardiovascular diseases (e.g., heart attack, stroke) |  |  |  |  |  |  |  |
| Cancer (e.g., breast cancer, lung cancer, colon cancer) |  |  |  |  |  |  |  |
| Diabetes mellitus |  |  |  |  |  |  |  |
| Chronic lung diseases (e.g., chronic obstructive pulmonary disease (COPD)) |  |  |  |  |  |  |  |
| Musculoskeletal disease (e.g., osteoporosis, osteoarthritis, back pain) |  |  |  |  |  |  |  |
| Dementia |  |  |  |  |  |  |  |

Question 6: Please rate how many deaths per 100 deaths occur due to which NCD in Switzerland. The sum of all numbers should add up to 100!

- Cardiovascular diseases (e.g., heart attack, stroke) _____
- Cancer (e.g., breast cancer, lung cancer, colon cancer) _____
- Diabetes mellitus _____
- Chronic lung diseases (e.g., COPD) _____
- Musculoskeletal disease (e.g., osteoporosis, osteoarthritis, back pain) _____
- Dementia _____
- Other causes of death (e.g. accidents, infections) _____

Total _____

Question 7: Do you think that it is possible to prevent the occurrence / progress of NCD?

- Yes
- No

Question 8: In your opinion, which of the following measures can prevent or delay the development and / or the progress of NCD?

|  |  | No, on the contrary | No | Rather no | Rather yes | Yes | I don’t know |
| --- | --- | --- | --- | --- | --- | --- | --- |
| 1. | Taking prescription medication |  |  |  |  |  |  |
| 2. | Being physically active daily |  |  |  |  |  |  |
| 3. | Maintaining a healthy diet |  |  |  |  |  |  |
| 4. | Eating with pleasure is more important than a rigid diet |  |  |  |  |  |  |
| 5. | Paying attention to the quality of food |  |  |  |  |  |  |
| 6. | Eating a lot of meat |  |  |  |  |  |  |
| 7. | Eating a lot of vegetables and fruits |  |  |  |  |  |  |
| 8. | Consuming a lot of sugar |  |  |  |  |  |  |
| 9. | Consuming a lot of salt |  |  |  |  |  |  |
| 10. | Drinking at least 1.5 l water everyday |  |  |  |  |  |  |
| 11. | Drinking alcohol everyday |  |  |  |  |  |  |
| 12. | Living in a non-smoking environment |  |  |  |  |  |  |
| 13. | Enjoying small amounts of alcohol sporadically |  |  |  |  |  |  |
| 14. | Living in the countryside |  |  |  |  |  |  |
| 15. | Being in nature |  |  |  |  |  |  |
| 16. | Stress reduction |  |  |  |  |  |  |
| 17. | Other: _____ |  |  |  |  |  |  |

*Comment: Oestrogens are protective against several NCD. Aging and menopause increase the prevalence of NCD in women. Accordingly, NCD mostly affect women 64+. The most killing NCD are cardiovascular diseases and cancer. In 100 women, 34 will die because of cardiovascular disease and 22 because of cancer. Yet, NCD are not only killing but reduce quality of life by, e.g., reduced mobility, chronic pain, depression.*

**Part B**

Most NCD are diagnosed at an advanced stage. Thus, it’s often impossible to completely heal the disease. If NCD are diagnosed at an early stage, treatment can help to prevent or delay progress. This is one reason, why so-called NCD risk calculators have been developed. Based on individual data NCD risk calculators estimate the personal risk to develop a NCD in the next 5-10 years.

Question 9: Have you ever heard about NCD risk calculators?

- Yes
- No

Question 10: If yes, where have you come across this term? (multiple answers possible)

- At my physician
- Family & friends
- Newspaper or (health) magazine
- Adds or flyers
- TV
- Internet
- School / other educational activities
- Other _____

Question 11: Has your physician (GP, gynaecologist, other) ever calculated your risk of developing a NCD or has (s)he offered you a NCD risk calculation?

- Yes
- No

Question 11b: If yes, which NCD has (s)he done the risk calculation for? (multiple answers possible)

- Cardiovascular diseases (e.g., heart attack, stroke)
- Cancer (e.g., breast cancer, lung cancer, colon cancer)
- Diabetes mellitus
- Chronic lung diseases (e.g., COPD)
- Musculoskeletal disease (e.g., osteoporosis, osteoarthritis, back pain)
- Dementia
- Others: _____

Question 12: For which NCD would you like your personal risk to be calculated? (multiple answers possible)

- Cardiovascular diseases (e.g., heart attack, stroke)
- Cancer (e.g., breast cancer, lung cancer, colon cancer)
- Diabetes mellitus
- Chronic lung diseases (e.g., COPD)
- Musculoskeletal disease (e.g., osteoporosis, osteoarthritis, back pain)
- Dementia
- Others: _____
- None

**Part C**

Public health care aims to reduce NCD burden on the individual as well as on the populational level. To achieve this goal the emphasis is laid on prevention. Accordingly, the individual’s knowledge about and responsibility for health maintenance are to be strengthened.

Question 13: In your opinion, to which extent would NCD development in affected people have been preventable by adopting a healthy lifestyle?

_____ %

Question 14: Imagine the following setting: You are healthy. You can prevent or delay the development of NCD. How willing are you to take the following measures?

|  | Completely willing | Possibly willing | Not willing | I’m already doing that |
| --- | --- | --- | --- | --- |
| Quit smoking |  |  |  |  |
| Quit alcohol drinking |  |  |  |  |
| Being physically active for 30 minutes per day (e.g., take the stairs, walk more) |  |  |  |  |
| Taking prescription medication(s) daily (possible side effects) |  |  |  |  |
| Healthy diet / nutrition counselling |  |  |  |  |
| Reducing sugary drinks |  |  |  |  |
| Mammogram every two years |  |  |  |  |
| Faecal occult blood test or colonoscopy |  |  |  |  |
| Attending stress management class |  |  |  |  |

Question 15: Second setting: You went to your GP for consultation. (S)he offered you to calculate your individual NCD risk. Your individual risk to develop a certain NCD turns out to be 30%. How willing are you to take the following measures?

*Comment: An individual risk of 30% means that one in three persons at the same age and with the same lifestyle and risks as you will develop a certain NCD (e.g., cancer, heart attack) in the next 5-10 years.*

|  | Completely willing | Possibly willing | Not willing | I’m already doing that |
| --- | --- | --- | --- | --- |
| Quit smoking |  |  |  |  |
| Quit alcohol drinking |  |  |  |  |
| Being physically active for 30 minutes per day (e.g., take the stairs, walk more) |  |  |  |  |
| Taking prescription medication(s) daily (possible side effects) |  |  |  |  |
| Healthy diet / nutrition counselling |  |  |  |  |
| Reducing sugary drinks |  |  |  |  |
| Mammogram every two years |  |  |  |  |
| Faecal occult blood test or colonoscopy |  |  |  |  |
| Attending stress management class |  |  |  |  |

Question 16: Third setting: You are now five years older and you’ve developed a certain NCD. How willing are you to take the following measures to slow down the progress of your disease and maintain your quality of life and independence?

|  | Completely willing | Possibly willing | Not willing | I’m already doing that |
| --- | --- | --- | --- | --- |
| Quit smoking |  |  |  |  |
| Quit alcohol drinking |  |  |  |  |
| Being physically active for 30 minutes per day (e.g., take the stairs, walk more) |  |  |  |  |
| Taking prescription medication(s) daily (possible side effects) |  |  |  |  |
| Healthy diet / nutrition counselling |  |  |  |  |
| Reducing sugary drinks |  |  |  |  |
| Mammogram every two years |  |  |  |  |
| Faecal occult blood test or colonoscopy |  |  |  |  |
| Attending stress management class |  |  |  |  |

Question 17: NCD risk calculators calculate your chance in % to develop a certain NCD within the next 5-10 years. Which % would be high enough for you to change your lifestyle?

*Comment: A 30% risk means that 30 in 100 persons at the same age and with the same lifestyle and risks will develop a certain NCD (e.g., cancer, heart attack) within the next 5-10 years.*

_____ %

Question 18: In your opinion, how is the availability of information on NCD prevention?

- Too much information
- Enough information
- Not enough information
- No information
- No opinion

Question 19: Which sources would you like to get information on NCD prevention from? (multiple answers possible)

- GP
- Health insurance
- Government
- Newspaper
- TV
- Radio
- School
- Others: _____
- No opinion

Question 20: What keeps you back from adopting a healthier lifestyle now? (multiple answers possible)

- Preparing healthy meals is too time-consuming
- Preparing healthy meals is too expensive
- A healthy diet isn’t really my taste
- Exercising is too time-consuming
- Exercising is too expensive
- Quitting smoking and/or drinking alcohol is difficult
- At the moment I have other issues to deal with that are more important to me
- I don’t know what to change
- I see any reason to change

Question 21: NCD cause more than 80% of direct health cost (= cost for diagnosis and treatment of a certain NCD, e.g., laboratory workup, drugs, hospitalization). In Switzerland, this corresponds to ca. 57 billion Swiss Francs per year. At the same time, public health care invests 2.2% of total cost (= ca. 1.5 billion Swiss Francs) in prevention.

In your opinion, which % of total cost should be invested in prevention? _____ %

- More money
- Exactly this amount of money
- Less money

*Comment: More than one in two persons could have avoided or at least delayed NCD onset by adopting a healthy lifestyle. Adopting a healthy lifestyle means: eating a balanced diet, being physically active on a regular basis, minimizing the intake of dependence causing substances and planning regular relaxation time. Furthermore, the body weight should be within the normal range. A healthy diet consists of at least 5 portions of fruits and vegetables per day, preferring whole-grain cereal, choosing healthy fats, limiting the sugar and salt intake and drinking enough non-sweetened beverages. Healthy fats can be found in nuts, fish or vegetable oil like olive or canola oil. The WHO recommends physical exercise for at least 30 minutes per day. Smoking, alcohol and illegal drugs are part of the dependence-causing substances that should be avoided. Adopting a healthy lifestyle is worthwhile. The earlier you start in life, the more powerful the effect becomes. Once the disease has manifested, it’s mostly not possible to treat it only with a healthy lifestyle. However, the disease progress can be slowed down. Therefore, a healthy lifestyle is also beneficial for people already suffering from NCD.*

**Part D**

Question 22: How old are you?

- 18 - 39 years
- 40 – 59 years
- 60 – 74 years
- ≥ 75 years

Question 23: What is your job occupation? (multiple answers possible)

- In training / student
- Working full-time
- Working part-time
- Housewife
- Retired

Question 24: What is your highest educational degree?

- Mandatory school
- Secondary Education (high school / specialized secondary school/ vocational school/ apprenticeship)
- Tertiary Education (university / technical college)
- Others: _____

Question 25: Do you have children?

- Yes
- No

Question 26: Is your health important to you?

- No opinion
- Not at all
- Moderately
- Very much

Question 27: Have you worried about your health status during the preceding month?

*Comment: Please enter your answer on the scale: 0 = I never worry, 10 = I worry very often*

| 0 | 1 | 2 | 3 | 4 | 5 | 6 | 7 | 8 | 9 | 10 |
| --- | --- | --- | --- | --- | --- | --- | --- | --- | --- | --- |

- No opinion

Question 28: What are your risks to develop NCD? (multiple answers possible)

- Active smoking
- Passive smoking
- > 1 glass of alcohol per day
- > 3 glasses of alcohol on occasions
- Diet mostly consists of fast food, sweets and / or little fruits and vegetables
- < 30 min exercise per day
- Elevated blood lipid levels / taking drugs to regulate elevated blood lipids
- Elevated blood pressure / taking drugs to regulate raised blood pressure
- Elevated blood sugar / taking drugs to regulate elevated blood sugar
- Others: _____

Question 29: What’s your body weight?

_____ kg

Question 30: What’s your body height?

_____ cm

Thank you for your interest and participation!

In the following section the correct answers are provided for the reader only (they were not shared with participants)

Correct answer to question 5: (1) (2)

*For all diseases were answers from moderately to severely accepted.*

*NCD disease burden measured in DALYs per 100’000 (total n=19'502) has been found to be highest for CVD (n=4282), followed by cancer (n=2736), musculoskeletal disorders (n=2462), diabetes and endocrine diseases (n=1777), chronic respiratory disease (n=1712), and neurological disorders (n=1071)*

Correct answer to question 6: (3)

- *Cardiovascular diseases (e.g., heart attack, stroke) 34%*
- *Cancer (e.g., breast cancer, lung cancer, colon cancer) 22%*
- *Diabetes mellitus 2%*
- *Chronic lung diseases (e.g., COPD) 5%*
- *Musculoskeletal disease (e.g., osteoporosis, osteoarthritis, back pain) not known*
- *Dementia 12%*
- *Other causes of death (e.g. accidents, infections) 25%*

*Total 100%*

Correct answer to question 7: (4) (5)

*Yes*

Correct answer to question 8: (6) (7) (8)

*Options 1, 2, 3, 5, 7, 10, 12, 16 are correct.*

*Options 8, 9 and 11 are wrong.*

*The rest of the answers don’t have a clear yes or no answer.*

Correct answer to question 14: (4) (5)

*Up to 50%*

**References:**

1. Van Dijk G. M., Kavousi M., Troup J., Franco O.W. Health issues for menopausal women: The top 11 conditions have common solutions. *Maturitas.* 80(1):24-30, 2015 Jan, https://doi: 10.1016/j.maturitas.2014.09.013.

2. Murray C.J.L., Vos T., Lozano R. et al. Disability-adjusted life years (DALYs) for 291 diseases and injuries in 21 regions, 1990-2010: a systematic analysis for the Global Burden of Disease Study 2010. *The Lancet.* 380(9859):2197-223, 2012 Dec 15, https://doi: 10.1016/S0140-6736(12)61689-4.

3. Bundesamt für Statistik BFS. Todesursachenstatistik Sterblichkeit und deren Hauptursachen in der Schweiz, 2016. *Schweizerische Eidgenossenschaft, Bundesamt für Statistik.* [Online] Neuchâtel 2019. https://www.swissstats.bfs.admin.ch/article/issue191412571600-01.

4. Koch U., de Falco A.A., von Greyerz S., Leutwyler S., Abel B. *Nationale Strategie Prävention nichtübertragbarer Krankheiten (NCD Strategie) 2017-2024.* s.l. : Bundesamt für Gesundheit und Schweizerische Konferenz der kantonalen Gesundheitsdirektorinnen und -direktoren, 2016.

5. World Health Organization, Regional Office for Europe. Health 2020 - A European policy framework and strategy for the 21st century. *World Health Organization.* [Online] 2013. http://www.euro.who.int/__data/assets/pdf_file/0011/199532/Health2020-Long.pdf?ua=1.

6. World Health Organization - Nutrition and Food Safety. Healthy diet. *World Health Organization.* [Online] 2018. http://www.who.int/mediacentre/factsheets/fs394/en/.

7. World Health Organization. Physical activity. *World Health Organization.* [Online] 2020. https://www.who.int/news-room/fact-sheets/detail/physical-activity.

8. World Health Organization. Alcohol. *World Health Organization.* [Online] 2018. https://www.who.int/news-room/fact-sheets/detail/alcohol.
